# Domain-Specific Pretraining of NorDeClin-Bidirectional Encoder Representations From Transformers for International Statistical Classification of Diseases, Tenth Revision, Code Prediction in Norwegian Clinical Texts: Model Development and Evaluation Study

**DOI:** 10.2196/66153

**Published:** 2025-08-25

**Authors:** Phuong Dinh Ngo, Miguel Ángel Tejedor Hernández, Taridzo Chomutare, Andrius Budrionis, Therese Olsen Svenning, Torbjørn Torsvik, Anastasios Lamproudis, Hercules Dalianis

**Affiliations:** 1 Norwegian Centre for E-health Research, University Hospital of Northern Norway, P.O. Box 35, N-9038, Tromsø, Norway, 47 92699162; 2 Department of Physics and Technology, Faculty of Sciences and Technology, UiT The Arctic University of Norway, Tromsø, Norway; 3 Department of Mathematics and Statistics, Faculty of Sciences and Technology, UiT The Arctic University of Norway, Tromsø, Norway; 4 Department of Computer Sciences, Faculty of Sciences and Technology, UiT The Arctic University of Norway, Tromsø, Norway; 5 Department of Computer and Systems Sciences, Stockholm University, Kista, Sweden

**Keywords:** natural language processing, artificial intelligence, language model, clinical text, BERT, text mining, health care, ICD-10 Coding

## Abstract

**Background:**

Accurately assigning *ICD-10* (*International Statistical Classification of Diseases, Tenth Revision*) codes is critical for clinical documentation, reimbursement processes, epidemiological studies, and health care planning. Manual coding is time-consuming, labor-intensive, and prone to errors, underscoring the need for automated solutions within the Norwegian health care system. Recent advances in natural language processing (NLP) and transformer-based language models have shown promising results in automating *ICD* (*International Classification of Diseases*) coding in several languages. However, prior work has focused primarily on English and other high-resource languages, leaving a gap in Norwegian-specific clinical NLP research.

**Objective:**

This study introduces 2 versions of NorDeClin-BERT (NorDeClin Bidirectional Encoder Representations from Transformers), domain-specific Norwegian BERT-based models pretrained on a large corpus of Norwegian clinical text to enhance their understanding of medical language. Both models were subsequently fine-tuned to predict ICD-10 diagnosis codes. We aimed to evaluate the impact of domain-specific pretraining and model size on classification performance and to compare NorDeClin-BERT with general-purpose and cross-lingual BERT models in the context of Norwegian ICD-10 coding.

**Methods:**

Two versions of NorDeClin-BERT were pretrained on the ClinCode Gastro Corpus, a large-scale dataset comprising 8.8 million deidentified Norwegian clinical notes, to enhance domain-specific language modeling. The base model builds upon NorBERT3-base and was pretrained on a large, relevant subset of the corpus, while the large model builds upon NorBERT3-large and was trained on the full dataset. Both models were benchmarked against SweDeClin-BERT, ScandiBERT, NorBERT3-base, and NorBERT3-large, using standard evaluation metrics: accuracy, precision, recall, and *F*_1_-score.

**Results:**

The results show that both versions of NorDeClin-BERT outperformed general-purpose Norwegian BERT models and Swedish clinical BERT models in classifying both prevalent and less common *ICD-10* codes. Notably, NorDeClin-BERT-large achieved the highest overall performance across evaluation metrics, demonstrating the impact of domain-specific clinical pretraining in Norwegian. These results highlight that domain-specific pretraining on Norwegian clinical text, combined with model capacity, improves *ICD-10* classification accuracy compared with general-domain Norwegian models and Swedish models pretrained on clinical text. Furthermore, while Swedish clinical models demonstrated some transferability to Norwegian, their performance remained suboptimal, emphasizing the necessity of Norwegian-specific clinical pretraining.

**Conclusions:**

This study highlights the potential of NorDeClin-BERT to improve *ICD-10* code classification for the gastroenterology domain in Norway, ultimately streamlining clinical documentation, reporting processes, reducing administrative burden, and enhancing coding accuracy in Norwegian health care institutions. The benchmarking evaluation establishes NorDeClin-BERT as a state-of-the-art model for processing Norwegian clinical text and predicting *ICD-10* coding, establishing a new baseline for future research in Norwegian medical NLP. Future work may explore further domain adaptation techniques, external knowledge integration, and cross-hospital generalizability to enhance *ICD* coding performance across broader clinical settings.

## Introduction

The transition to digital health records and the automation of clinical documentation processes represent significant milestones in modern health care management. Central to these advancements is the accurate assignment of the *ICD-10* (*International Statistical Classification of Diseases, Tenth Revision*) codes to patient records [1]. These codes serve multiple critical functions: they streamline billing and insurance claims, play a pivotal role in epidemiological studies, facilitate health care planning, and aid in the management of public health resources. In addition, they serve as a measure of both the quantity and quality of health care provided [2].

In Norway, all hospitals record their activity by summarizing patient encounters into *ICD-10* codes. Despite its importance, manually assigning *ICD-10* codes is time-consuming and prone to errors, highlighting the need for an automatic solution [3,4]. Several Norwegian studies have highlighted the issues associated with clinical coding [5-7], and similar findings from other countries support this nonsatisfactory quality of the manually assigned codes [8-11].

Recent advances in natural language processing (NLP), particularly the development of Bidirectional Encoder Representations from Transformers (BERT) models [12], have facilitated novel methodologies for automating complex text data processing. Specifically, the architecture of the BERT transformer facilitates a good understanding of contextual linguistic nuances, making it highly applicable for various clinical tasks, including deidentification [13,14] and the prediction of *ICD-10* codes from clinical notes [15]. Building on these advancements, NorBERT3-base, developed by the Language Technology Group [16] at the University of Oslo and available on Hugging Face [17], is an advanced, state-of-the-art Norwegian BERT model tailored to understand the complexities of the Norwegian language. It was trained as part of the NorBench initiative, which benchmarks Norwegian language models across various NLP tasks to ensure high performance and robustness. NorBERT3-base is a powerful tool for NLP tasks such as text classification and named entity recognition [18].

Regarding the state-of-the-art automatic *ICD* (*International Classification of Diseases*) coding or computer-assisted coding (CAC) tools, Yan et al [19] provided an overview of different approaches to predict *ICD-10* diagnosis codes, describing training datasets in various languages and highlighting issues such as dataset imbalance and explainability. Studies from China [20] and Taiwan [21] have demonstrated the potential of these tools to improve coding speed and quality.

In the study by Zhou et al [20], a set of regular expressions was written to encode the diagnosis of *ICD-10* automatically. The CAC tool was used for 16 months in 2017‐2018 and compared with manual diagnosis coding. During this period, 160,000 codes were automatically assigned by the CAC tool and then compared with the manual coding. One of the main findings was that the CAC tool was 100 times faster than manual coding, and the CAC tool could maintain high coding quality. The *F*_1_-score of the CAC tool is around 0.6086. In another study by Chen et al [21], the authors implemented a CAC tool using the BERT model. They trained on patient records from one hospital. A total of 14,602 labels were distributed in the training material that comprised discharge summaries. Note that Chinese and Taiwanese use the same dialect but have different character sets, which are simplified and traditional. The Taiwanese *ICD-10* CAC tool predicts *ICD-10* codes with the best results of *F*_1_-score of 0.715 and 0.618, respectively. The tool was also used in a user study that did not decrease coding time; however, the coding quality increased significantly from a median *F*_1_-score of 0.832 to 0.922. Ponthongmak et al [22] used NLP and discharge summary texts to develop a CAC tool for Thai, achieving an *F*_1_-score of 0.7239 using a pretrained language model for automatic *ICD* coding. A systematic literature review of artificial intelligence (AI)–based *ICD* coding and classification approaches using discharge summaries can be found in [23].

Several studies have also explored pretraining on clinical text for automatic *ICD* coding, leveraging transformer-based architectures. One such approach is GPsoap, developed by Yang et al [24], which transforms *ICD* coding into an autoregressive text generation task. Instead of directly predicting *ICD* codes, GPsoap first generates natural language code descriptions, which are then mapped to *ICD* codes. This approach has shown advantages in few-shot learning and rare code prediction. Other studies, such as López-García et al [25], focused on Spanish oncology clinical texts, where a BERT-based model was pretrained on Spanish biomedical literature and further fine-tuned on ICD-O-3 (International Classification of Diseases for Oncology) coding. Similarly, Gao et al [26] proposed BNMF, a BERT and Named Entity Recognition-based model for Chinese *ICD* coding, integrating semantic features from clinical text and structured information from *ICD* taxonomies. These studies highlight the importance of domain-specific adaptation when applying language models to clinical coding.

In contrast, our approach focuses on pretraining a domain-specific clinical BERT model, NorDeClin-BERT, directly on Norwegian clinical text, enabling robust *ICD-10* classification in a multilabel setting. Unlike GPsoap, which generates free-text descriptions, our model directly assigns *ICD-10* codes, aligning more closely with real-world coding workflows. Additionally, our work explores cross-linguistic transfer, evaluating models pretrained on Swedish clinical text for Norwegian *ICD* coding. By fine-tuning various general-domain and domain-specific Scandinavian BERT models, we systematically assess the impact of domain adaptation, model size, and linguistic generalization on *ICD* coding performance.

Furthermore, our approach provides a comprehensive evaluation of model performance across both domain-adapted and general-purpose pretraining approaches, offering insights into the effectiveness of Norwegian-specific pretraining compared with multilingual and cross-lingual alternatives. By investigating how domain-specific pretraining influences *ICD-10* coding accuracy, our study contributes to advancing automatic clinical coding for Norwegian, a language with limited prior research in this area. While model performance is crucial, interpretability is equally important, especially in health care settings where understanding the reasoning behind predictions can impact patient care and trust in the system. Various approaches to model interpretability have been explored in the context of automated *ICD-10* coding. For example, Dolk et al [27] evaluated 2 popular interpretability methods, LIME (Local Interpretable Model-agnostic Explanations) and SHAP (Shapley additive explanations), to explain automatic *ICD-10* classifications of Swedish gastrointestinal discharge summaries, where SHAP was considered better than LIME. In our study, we opted for an attention-based analysis instead of LIME or SHAP. This choice is motivated by several factors. Attention mechanisms are inherent to BERT and other Transformer-based models, providing a direct window into the model’s decision-making process without requiring post hoc explanations. Furthermore, attention-based interpretability can be extracted during inference, making it more computationally efficient than methods like LIME and SHAP. Attention weights offer fine-grained, token-level insights into which parts of the input text the model focuses on when making predictions, aligning well with the nature of the clinical text and *ICD-10* coding tasks.

Our research group has previously explored the application of NLP techniques to improve the accuracy and efficiency of *ICD-10* diagnosis coding. In a recent study, we developed a BERT-based language model, SweDeClin-BERT, trained on a large open clinical corpus of Swedish discharge summaries, particularly in the gastrointestinal surgery domain [13]. This model demonstrated significant potential in assigning *ICD-10* codes to discharge summaries written in Swedish [15]. Building on the insights gained from this work, we have extended our focus to the Norwegian clinical context, aiming to develop a specialized language model tailored to the nuances of Norwegian medical texts.

This study introduces 2 versions of NorDeClin-BERT, BERT-based models specifically developed and fine-tuned for processing Norwegian clinical texts and predicting *ICD-10* codes. We detail the continuous pretraining process of NorDeClin-BERT-base from NorBERT3-base using a large, relevant subset of Norwegian gastroenterological clinical notes, and NorDeClin-BERT-large from NorBERT3-large using the full clinical corpus. By leveraging domain-specific pretraining on Norwegian clinical texts, both models capture the unique linguistic features and domain-specific terminology in Norwegian medical documentation. To assess their effectiveness, we compared the performance of NorDeClin-BERT with other BERT variants, including ScandiBERT [28] and NorBERT [29]. This comparative analysis aims to provide insight into the advantages of a domain-specific, language-tailored model for Norwegian clinical text processing.

To guide our study, we defined the following research questions (RQs):

- RQ1: Does domain-specific pretraining on Norwegian clinical text improve *ICD-10* code classification performance compared with general-domain and cross-lingual models?
- RQ2: How does model size impact performance in *ICD-10* coding tasks when combined with clinical domain adaptation?
- RQ3: Can a domain-specific base-size model match or outperform larger general-purpose models in a practical clinical classification task?

## Methods

### Overview

This study adopts a structured approach to the continuous pretraining and evaluation of 2 NorDeClin-BERT, new clinical BERT-based models developed for predicting *ICD-10* codes from Norwegian clinical notes, specifically focusing on the gastroenterology domain. This section covers ethical considerations related to data use, the process of data collection and preparation, selection of model architecture and continuous pretraining, fine-tuning, evaluation, and interpretability analysis.

### Ethical Considerations

This research was approved by the Norwegian Regional Committees for Medical and Health Research Ethics North, decision number 260972. This study is based on a retrospective analysis of deidentified clinical text. The ethics committee granted a waiver of informed consent in accordance with Norwegian regulations for secondary use of health data in research. All data used in this study were fully deidentified prior to analysis. No personal identifiers were included in the dataset. Access to the data was restricted to authorized personnel, and all analyses were conducted in secure computing environments. No compensation was provided to individuals, as the study did not involve direct participation and was conducted on retrospective clinical data. The manuscript does not contain any images or materials in which individual participants or users can be identified. All data used in this study were fully deidentified prior to analysis. No personal identifiers were included in the dataset. Access to the data was restricted to authorized personnel, and all analyses were conducted in secure computing environments.

All data used in this study were fully deidentified prior to analysis. No personal identifiers were included in the dataset. Access to the data was restricted to authorized personnel, and all analyses were conducted in secure computing environments.

### Dataset and Data Processing

#### Overview

The corpus for this study, the ClinCode Gastro Corpus, contains approximately 8.8 million deidentified and pseudonymized clinical notes [30] of adult patients treated at the Gastro-Surgical Department of the University Hospital of North Norway, Tromsø, from 2017 to 2022. The dataset was subjected to rigorous preprocessing, including deidentification using the NorDeid tool, to ensure patient privacy and data quality [30]. The NorDeid tool combines deep learning and rule-based approaches using regular expressions. This tool was adapted for the Norwegian clinical text to address the country’s unique format and clinical terminology. The process involved identifying and pseudonymizing various protected health information types, such as names, dates, locations, and social security numbers.

We used the tokenizer associated with each corresponding backbone model during preprocessing: the NorBERT3-base tokenizer for NorDeClin-BERT-base, and the NorBERT3-large tokenizer for NorDeClin-BERT-large. Trained on a general corpus of Norwegian text, these tokenizers effectively handle the linguistic characteristics of the Norwegian language through their subword tokenization technique. Although not specifically constructed for clinical terminology, their subword approach allowed them to manage specialized medical terms and abbreviations present in our dataset during the continuous pretraining phase.

#### Data Processing for Continuous Pretraining

Two configurations of NorDeClin-BERT were pretrained using Norwegian clinical notes, each with a different data selection strategy. For NorDeClin-BERT-base, the dataset was filtered based on clinical relevance and practical feasibility, due to limited computational resources available at the time. Two of the authors of this paper (MATH and TOS) collaborated to identify and agree on the most informative files for the pretraining process. The selection criteria focused on document types containing longer and more meaningful clinical information, ensuring the model was pretrained on the most relevant data. As a result, the final dataset used for pretraining was optimized for both quality and relevance. After removing duplicates, the Norwegian clinical corpus used for the continuous pretraining of NorDeClin-BERT-base consisted of 1,670,464 text files (3.2 GB) from various sources, including discharge summaries, surgery notes, nurses’ notes, laboratory notes, admission notes, pharmacology notes, and others. This dataset is further described in Table 1.

**Table 1.** Document types included in the Norwegian clinical corpus used for the continuous pretraining of NorDeClin-BERT-base (NorDeClin Bidirectional Encoder Representations from Transformers).

Document type	Number of files	Size
Anesthesia	46,310	94.8 MB
Treatment	29,919	49.3 MB
Discharge summaries	586,637	1.6 GB
Ergotherapy	33,220	38.4 MB
Pharmacy	3484	4.6 MB
Physiotherapy	69,324	80.4 MB
Individual plan	558	1.4 MB
Admission records	248,208	779,2 MB
Laboratory	66	53.8 kB
Surgery	313,795	446.8 MB
Summary records	5710	9.2 MB
Radiology	63,734	30.1 MB
Somatic care	110,248	211.3 MB
Nursing	299,212	220.7 MB
Training dataset (no duplicates)	1,670,464	3.2 GB

In contrast, NorDeClin-BERT-large was pretrained on the entire ClinCode Gastro Corpus, using updated hardware infrastructure with increased graphics processing unit (GPU) capacity. The only filtering applied at this stage was the removal of very short documents, excluding those with fewer than 50 tokens, to ensure a minimum level of linguistic and contextual content per note. After filtering, the dataset used for NorDeClin-BERT-large consisted of 8,337,664 text files, totaling approximately 13.2 GB. This broader dataset allowed the large model to capture a more comprehensive representation of the Norwegian clinical language used across document types.

The data processing pipeline for continuous pretraining began with loading and reading the text files from the specified directory. We applied the appropriate tokenizer to convert the text into token IDs while generating the corresponding attention masks. The text was processed in chunks, each constrained to a sequence length of 512 tokens, ensuring compatibility with the model’s architecture.

Several key steps were involved in preparing the text data. Initially, empty lines and whitespace were removed, followed by tokenization without adding special tokens. We then introduced separation tokens (</s>), as recommended in the RoBERTa paper [31], to demarcate the end of individual documents within the text. After concatenating the tokenized text into segments of 510 tokens—leaving space for the model’s classification (<s>) and separator (</s>) tokens—we added these tokens to the beginning and end of each segment, enabling the model to recognize the start and end of sequences effectively. Finally, the processed data was saved to disk in a structured format, ready for continuous pretraining.

#### Data Processing for *ICD-10* Fine-Tuning

The *ICD-10* is a standardized system for coding diseases, signs, symptoms, and other health-related factors. The *ICD-10* is divided into 22 chapters, each representing a broad category of medical conditions. Our study focuses specifically on Chapter XI (K-codes), which covers “Diseases of the digestive system.” This chapter contains approximately 500 “K” codes representing various gastrointestinal diseases out of the 38,000 *ICD-10* codes available. The presence of 87,938 discharge summaries with K-codes in our corpus underscores the richness and relevance of our dataset for gastroenterological research and NLP applications in this field. Furthermore, to prevent label leakage during the fine-tuning process, all *ICD-10* codes that match the label for each training sample were systematically removed from the training text. This step was essential to ensure the model learns to predict *ICD-10* codes based on clinical content rather than relying on explicitly mentioned codes within the text.

### Model Continuous Pretraining

This study presents 2 versions of NorDeClin-BERT, both developed through continuous pretraining on Norwegian clinical text. The first, NorDeClin-BERT-base, is based on the NorBERT3-base architecture, consisting of 12 transformer layers, 12 self-attention heads per layer, and a hidden size of 768 dimensions, resulting in 123 million parameters. The second, NorDeClin-BERT-large, builds upon NorBERT3-large, which features 24 layers, 16 attention heads per layer, a hidden size of 1024 dimensions, and approximately 340 million parameters. These architectures were selected for their proven effectiveness in capturing contextual information and learning rich language representations.

While both NorDeClin-BERT models retain the architecture of their respective backbone models, they differ in the domain-specific knowledge acquired through further pretraining. NorBERT3-base and NorBERT3-large were originally pretrained on general-domain Norwegian text, whereas we further trained both models on deidentified and pseudonymized Norwegian clinical text to create NorDeClin-BERT-base and NorDeClin-BERT-large, respectively. This continuous pretraining process enhanced the models’ ability to understand and represent medical language more effectively, making them well-suited for downstream clinical tasks such as *ICD-10* code prediction.

To further pretrain the NorDeClin-BERT models on our specialized clinical text data, we used a well-structured training pipeline built upon the Hugging Face Transformers library [32]. The pretraining process was carried out on a Republic of Gamers server running Debian Linux, initially equipped with a single ASUS GeForce RTX 3090 GPU and later expanded to support dual GPUs for training larger configurations. The system has 64 GB of RAM (2×32GB 3200 MHz DDR4), and an 8 TB Gen4×4 M.2 NVMe SSD. The server storage was encrypted and located in a secure server room, accessible only to researchers who were specially authorized to work with the data and had signed confidentiality agreements. The server was not connected to the internet to ensure data security and remained offline throughout the project.

NorDeClin-BERT was continuously pretrained using the masked language modeling (MLM) objective. In MLM, a portion of the input tokens is randomly masked, and the model is trained to predict the original tokens based on the surrounding context. This approach allows the model to learn robust representations of words and their relationships. Following the findings from the RoBERTa paper [31], which indicated that the next-sentence prediction task was unnecessary, we opted to focus exclusively on MLM during the pretraining of both versions of NorDeClin-BERT.

The tokenized data parts were loaded and concatenated to form a complete training dataset. The dataset was designed to be dynamically masked during training, where tokens were randomly masked at a probability of 15% to train the model on the MLM objective. Training parameters were carefully configured to optimize the model’s performance, closely following the RoBERTa paper [31]. Both NorDeClin-BERT-base and NorDeClin-BERT-large were pretrained for 40 epochs with a learning rate of 0.0001.

For NorDeClin-BERT-base, the batch size was configured for 8 sequences per device, with gradient accumulation steps set to 16, effectively simulating a larger batch size of 128 sequences. For NorDeClin-BERT-large, the configuration was adapted for dual-GPU training, using a batch size of 16 and accumulation steps of 2 per device, yielding an effective batch size of 64. While not identical, these settings were selected to maintain stable training dynamics under different hardware constraints. Additionally, the training process included a warmup phase (10,000 steps for base, 5000 for large), weight decay of 0.01, and no gradient clipping. The Adam optimizer was used with custom ε of 0.000001, β_1_ of 0.9, and β_2_ of 0.999. During the training process, checkpoints were saved periodically, with retention limits in place to manage disk space efficiently. The training could be resumed from a specific checkpoint if needed.

### Fine-Tuning

**Figure 1.**
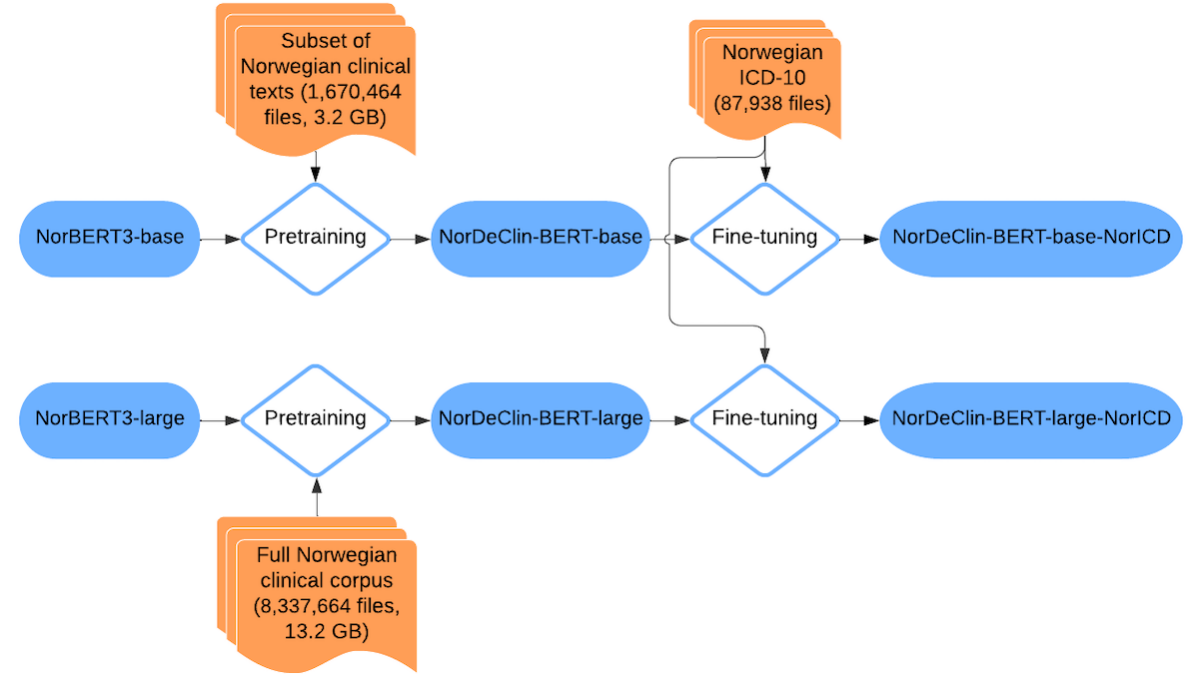
Workflow of the NorDeClin-BERT models. The models are initialized from NorBERT3-base and NorBERT3-large and further pretrained on Norwegian clinical texts to create NorDeClin-BERT-base and NorDeClin-BERT-large. Both are then fine-tuned on the Norwegian *ICD-10* coding task, resulting in the specialized classification models NorDeClin-BERT-base-NorICD and NorDeClin-BERT-large-NorICD. BERT: Bidirectional Encoder Representations from Transformers; *ICD-10*: *International Statistical Classification of Diseases, Tenth Revision*.

After continuous pretraining, both NorDeClin-BERT-base and NorDeClin-BERT-large were fine-tuned for *ICD-10* code prediction using 87,938 discharge summaries with K-codes. The dataset was partitioned into training (70,350/87,938, 80%), validation (8794/87,938, 10%), and testing (8794/87,938, 10%) sets. The fine-tuning process began with data preparation, where the discharge summaries and their corresponding *ICD-10* codes were loaded from a CSV file using the Hugging Face datasets library. Each summary’s codes were split into a list format for further processing. Figure 1 illustrates this workflow, showing how the NorDeClin-BERT models were pretrained on Norwegian clinical texts and subsequently fine-tuned on the Norwegian *ICD-10* coding task to create the final classification models.

A custom preprocessing function was implemented to tokenize the text and prepare the labels. This function tokenized each discharge summary using the model’s tokenizer with a maximum sequence length of 512 tokens. Labels were encoded using a multihot encoding scheme, where each unique *ICD-10* code was represented as a binary vector. The NorDeClin-BERT models were then loaded with a classification head adapted for multilabel classification, with the number of output labels set to match the total number of unique *ICD-10* codes in the dataset.

The training setup used a custom MultilabelTrainer class, extending the HuggingFace Trainer class for multilabel classification. The trainer used a binary cross-entropy loss function BCEWithLogitsLoss and was configured with specific hyperparameters: 40 epochs, a learning rate of 2e-5, and an early stopping patience of 1 epoch. To effectively manage memory constraints and increase the batch size, the training used a batch size of 4 with 16 gradient accumulation steps, resulting in a batch size of 64.

During the fine-tuning process, the model was trained on the prepared dataset, with evaluation performed on the validation set after each epoch. Early stopping was applied to prevent overfitting, and the best model was saved based on validation performance. The training process used a constant learning rate scheduler.

After training, the models were evaluated on the held-out test set using custom metric functions to compute accuracy, precision, recall, and *F*_1_-score for multilabel classification. A threshold of 0.5 was applied to the model’s output probabilities to determine the final predictions.

### Evaluation and Benchmarking of the NorDeClin-BERT Models

#### Overview

**Figure 2.**
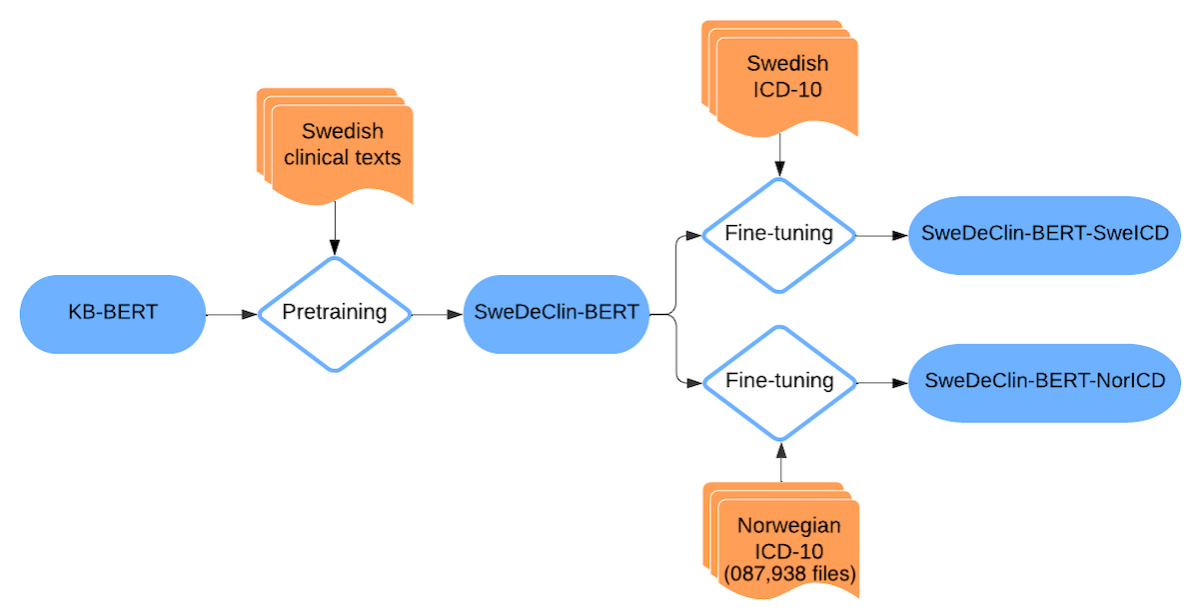
Workflow of the SweDeClin-BERT model. The model is initialized from KB-BERT and further pretrained on Swedish clinical texts. It is then fine-tuned separately on Swedish and Norwegian *ICD-10* coding tasks, resulting in 2 specialized versions: SweDeClin-BERT-SweICD and SweDeClin-BERT-NorICD. BERT: Bidirectional Encoder Representations from Transformers; *ICD-10*: *International Statistical Classification of Diseases, Tenth Revision*.

**Figure 3.**
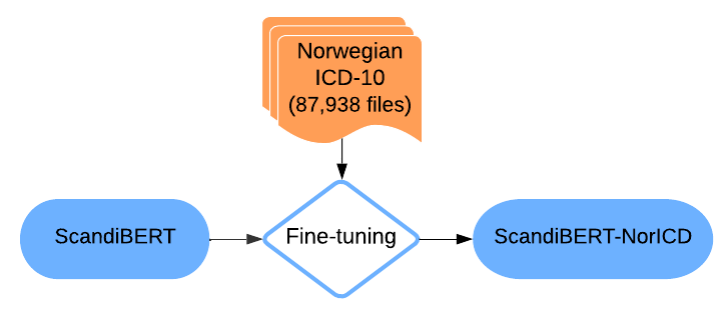
Workflow of the ScandiBERT model. The model is initialized from ScandiBERT and fine-tuned on the Norwegian *ICD-10* coding task. BERT: Bidirectional Encoder Representations from Transformers; *ICD-10*: *International Statistical Classification of Diseases, Tenth Revision*.

**Figure 4.**
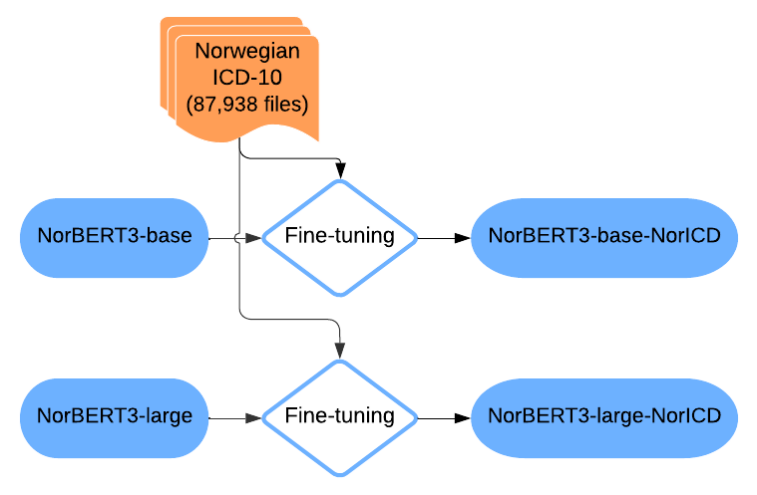
Workflow of the NorBERT3 models. The base and large variants of NorBERT3 are fine-tuned on the Norwegian *ICD-10* coding task. This results in 2 specialized models: NorBERT3-base-NorICD and NorBERT3-large-NorICD. BERT: Bidirectional Encoder Representations from Transformers; *ICD-10: International Classification of Diseases, Tenth Revision*.

To benchmark the NorDeClin-BERT models’ performance, we carefully selected several other BERT-based models for comparison. Each model was chosen to provide specific insights into different aspects of language modeling and transfer learning in the context of Scandinavian languages and clinical text processing. Norwegian and Swedish, as closely related North Germanic languages, share significant lexical, syntactic, and morphological similarities, making cross-linguistic model transfer feasible. Medical terminology is also largely standardized across Scandinavian countries, further supporting the applicability of models trained on one language to another. Given these linguistic and domain-specific similarities, evaluating the NorDeClin-BERT models against models trained on Swedish and general-domain Scandinavian corpora provides valuable insights into how well these models generalize within the Nordic clinical context. To better illustrate the methodological differences across models, we provide individual workflow diagrams (Figures 1-4) and a summary table (Table 2), which highlight variations in model pretraining, fine-tuning, and dataset composition, facilitating direct comparison.

**Table 2.** Overview of the models used in the study. The table presents each model’s size, type, pretraining data, and fine-tuning task.

Model	Size	Type	Pretrained from	Pretraining	Fine-tuning
NorDeClin-BERT-base-NorICD^a^^b^	Base	Domain-Specific Clinical BERT	NorBERT3-base	Subset of Norwegian clinical texts	Norwegian *ICD-10*
SweDeClin-BERT-SweICD	Base	Domain-Specific Clinical BERT	KB-BERT	Swedish clinical texts	Swedish *ICD-10*
SweDeClin-BERT-NorICD	Base	Domain-Specific Clinical BERT	KB-BERT	Swedish clinical texts	Norwegian *ICD-10*
ScandiBERT-NorICD	Base	General-Domain BERT	—^c^	No	Norwegian *ICD-10*
NorBERT3-base-NorICD	Base	General-Domain BERT	—	No	Norwegian *ICD-10*
NorDeClin-BERT-large-NorICD	Large	Domain-Specific Clinical BERT	NorBERT3-large	Full Norwegian clinical corpus	Norwegian *ICD-10*
NorBERT3-large-NorICD	Large	General-Domain BERT	—	No	Norwegian *ICD-10*

a BERT: Bidirectional Encoder Representations from Transformers.

b *ICD-10*: *International Statistical Classification of Diseases, Tenth Revision*.

c Not applicable.

The following sections contain a detailed explanation of each model and the rationale behind its inclusion.

#### SweDeClin-BERT

This model originates from KB-BERT [33], a standard Swedish BERT model, which was further pretrained on Swedish clinical texts [13]. It uses the base BERT architecture with 12 layers, 768 hidden units, and 12 attention heads, totaling approximately 110 million parameters. This comparison helps evaluate the importance of language-specific training in clinical NLP tasks. SweDeClin-BERT is represented by 2 variants in our evaluation task: SweDeClin-BERT-SweICD and SweDeClin-BERT-NorICD. SweDeClin-BERT-SweICD is a variant of SweDeClin-BERT, which was further fine-tuned on Swedish datasets for *ICD-10* code classification. SweDeClin-BERT-NorICD represents SweDeClin-BERT further fine-tuned on the Norwegian ClinCode Gastro Corpus. Their inclusion allows us to assess the performance of models specifically designed for clinical text but in a closely related Scandinavian language. By comparing their performance with the NorDeClin-BERT models, we can also determine how well clinical knowledge and *ICD-10* classification capabilities transfer from Swedish to Norwegian. Figure 2 illustrates the training and fine-tuning process of SweDeClin-BERT, highlighting its pretraining on Swedish clinical text and subsequent fine-tuning for *ICD-10* classification in both Swedish and Norwegian.

#### ScandiBERT

A model [34] pretrained on a mix of Scandinavian languages designed to capture the linguistic characteristics of the region [28]. Its inclusion allows for evaluating the effectiveness of a multilingual model compared with a language-specific model in the *ICD-10* coding prediction task. Figure 3 illustrates the fine-tuning process of ScandiBERT, where the model is adapted to Norwegian clinical text using *ICD-10* coding data, resulting in the ScandiBERT-NorICD variant.

#### NorBERT3 (Base and Large Variants)

A model developed for the Norwegian language [29]. NorBERT3-base uses a similar architecture to the other base models, while NorBERT3-large uses a larger architecture with 24 layers, 1024 hidden units, and 16 attention heads, totaling approximately 340 million parameters. Including both base and large variants allows assessing the impact of model size on performance. Additionally, comparing these general-domain models with the NorDeClin-BERT-base and NorDeClin-BERT-large models provides a fair assessment of the effects of clinical domain adaptation versus general language pretraining for Norwegian. Figure 4 illustrates the fine-tuning process of NorBERT3-base and NorBERT3-large on Norwegian *ICD-10* coding tasks, resulting in the specialized models NorBERT3-base-NorICD and NorBERT3-large-NorICD.

#### Evaluation Metrics

##### Overview

Each model was fine-tuned and evaluated using the same training, validation, and testing splits of the dataset. We used a comprehensive evaluation strategy focusing on the following metrics: accuracy, precision, recall, and *F*_1_-score [35]. Accuracy measures the proportion of correct predictions out of the total predictions made, providing an overall effectiveness of the model. Precision indicates the proportion of true positive predictions among all positive predictions, where high precision means that the model has a low false-positive rate. The recall represents the proportion of true positive predictions among all actual positives, with high recall indicating the model’s ability to identify most of the relevant instances. The *F*_1_-score, as the harmonic mean of precision and recall, provides a single metric that balances both concerns, which is particularly useful when the class distribution is imbalanced. These metrics were calculated considering the multilabel nature of the problem using weighted averages. The evaluation was carried out for both the complete set of codes and the top 80% codes that are used the most. We applied these metrics in 2 main evaluation strategies: multilabel evaluation and top-5 evaluation.

##### Multilabel Evaluation

Given the multilabel nature of *ICD-10* coding, where multiple codes may apply to a single clinical note, we analyzed model performance in predicting the exact set of relevant codes at the sample level. This was achieved by converting the model’s output logits to probability scores and applying a threshold of 0.5 to generate binary predictions, where a label is considered predicted if its probability is greater than or equal to 0.5. These binary predictions were then compared against the true labels to compute accuracy, precision, recall, and *F*_1_-score, providing a detailed view of the model’s ability to handle multiple simultaneous labels correctly.

##### Top-5 Evaluation

This evaluation assesses the model’s ability to predict the top-5 most probable codes for each clinical note at the sample level, reflecting practical coding scenarios where identifying the most relevant codes quickly is crucial. The process involved sorting the probability scores for each sample to identify the top 5 highest scoring labels and converting these indices to their corresponding *ICD-10* codes. The actual labels present in the ground truth were then extracted for each sample. Each actual label was checked to see if it was among the top 5 predicted labels. If the actual label was among the top 5 predicted labels, it was added to both the actual and predicted lists. If not, the actual label was added to the actual list, and the last element in the top-5 predictions was added to the predicted list. Finally, the evaluation metrics, including accuracy, precision, recall, and *F*_1_-score, were calculated by comparing the predicted and label lists.

### Model Interpretability

To provide insights into the decision-making processes of the NorDeClin-BERT models, an attention-based interpretability analysis was conducted. This involved generating a synthetic clinical text using ChatGPT, processing the text through both NorDeClin-BERT-base-NorICD and NorDeClin-BERT-large-NorICD models, extracting attention weights, aggregating and normalizing attention weights across all layers and heads, and visualizing attention distribution across input tokens during *ICD-10* code prediction.

This methodology allows for a comprehensive evaluation of the NorDeClin-BERT models’ performance, their comparative advantages over other BERT variants, and insights into their internal decision-making processes, all crucial for assessing their potential in automating *ICD-10* coding in Norwegian health care settings.

## Results

### Model Performance

The evaluation of the NorDeClin-BERT models and their comparison with other BERT-based models across 4 critical metrics (accuracy, precision, recall, and *F*_1_-score) yielded meaningful insights into their performance on *ICD-10* code classification tasks. The analysis was conducted for 2 distinct scenarios: classification performance for all codes and the top 80% most frequently used codes. Performance was further categorized into multilabel and top-5 accuracy.

### Accuracy

Table 3 presents the accuracy scores across all evaluated models under 4 evaluation settings. NorDeClin-BERT-large-NorICD achieved the highest accuracy across all scenarios, including multilabel (0.47) and top-5 (0.82) classification of the full *ICD-10* code set, as well as multilabel (0.56) and top-5 (0.88) classification of the top 80% most-used codes. It outperformed all other models, including the larger general-domain NorBERT3-large-NorICD, with the largest margin observed in the all codes multilabel setting (0.47 vs 0.42).

**Table 3.** Comparison of the accuracy of different BERT (Bidirectional Encoder Representations from Transformers) models.

Model size and model name	All codes, 95% CI		Top 80% codes, 95% CI	
	Multilabel	Top-5	Multilabel	Top-5
Base				
NorDeClin-BERT-base-NorICD^a^	0.44 (0.43-0.45)	0.81 (0.80-0.81)	0.54 (0.53-0.55)	0.87 (0.86-0.88)
SweDeClin-BERT-SweICD	0.25 (0.24-0.26)	0.59 (0.58-0.60)	0.35 (0.34-0.36)	0.65 (0.63-0.65)
SweDeClin-BERT-NorICD	0.40 (0.39-0.41)	0.78 (0.77-0.79)	0.50 (0.49-0.51)	0.85 (0.84-0.86)
ScandiBERT-NorICD	0.39 (0.38-0.40)	0.78 (0.77-0.79)	0.51 (0.50-0.52)	0.85 (0.84-0.86)
NorBERT3-base-NorICD	0.43 (0.42-0.44)	0.80 (0.79-0.81)	0.52 (0.51-0.53)	0.86 (0.86-0.87)
Large				
NorDeClin-BERT-large-NorICD	0.47 (0.46-0.48)^b^	0.82 (0.82-0.83)^b^	0.56 (0.55-0.57)^b^	0.88 (0.88-0.89)^b^
NorBERT3-large-NorICD	0.42 (0.41-0.43)	0.81 (0.80-0.82)	0.53 (0.52-0.54)	0.88 (0.87-0.88)^b^

a *ICD-10*: *International Statistical Classification of Diseases, Tenth Revision*.

b Highest score for each scenario.

Among base-sized models, NorDeClin-BERT-base-NorICD also showed strong performance, surpassing SweDeClin-BERT-NorICD, ScandiBERT-NorICD, and SweDeClin-BERT-SweICD in all settings. Notably, it matched or exceeded the performance of the larger NorBERT3-large-NorICD in 3 out of 4 scenarios, highlighting the impact of clinical domain adaptation even in smaller models.

### Precision

Table 4 presents precision scores across all models and evaluation scenarios. NorDeClin-BERT-large-NorICD achieved the highest precision in 3 out of 4 settings, including all codes multilabel (0.66), top-5 (0.82), and top 80% most-used codes top-5 (0.90). It performed comparably to NorBERT3-large-NorICD in the remaining setting, where NorBERT3-large-NorICD achieved a higher top 80% most-used codes multilabel precision (0.73 vs 0.72).

**Table 4.** Comparison of the precision of different BERT (Bidirectional Encoder Representations from Transformers) models.

Model size and model name	All codes, 95% CI		Top 80% codes, 95% CI	
	Multilabel	Top-5	Multilabel	Top-5
Base				
NorDeClin-BERT-base-NorICD^a^	0.65 (0.64-0.66)	0.80 (0.79-0.81)	0.71 (0.70-0.73)	0.89 (0.88-0.90)
SweDeClin-BERT-SweICD	0.38 (0.36-0.40)	0.61 (0.60-0.62)	0.46 (0.44-0.48)	0.69 (0.67-0.70)
SweDeClin-BERT-NorICD	0.58 (0.56-0.59)	0.77 (0.76-0.78)	0.66 (0.65-0.68)	0.87 (0.86-0.88)
ScandiBERT-NorICD	0.57 (0.55-0.58)	0.77 (0.76-0.78)	0.67 (0.66-0.69)	0.87 (0.87-0.88)
NorBERT3-base-NorICD	0.63 (0.61-0.64)	0.79 (0.78-0.80)	0.69 (0.68-0.70)	0.88 (0.88-0.89)
Large				
NorDeClin-BERT-large-NorICD	0.66 (0.65-0.68)^b^	0.82 (0.81-0.82)^b^	0.72 (0.71-0.74)	0.90 (0.90-0.91)^b^
NorBERT3-large-NorICD	0.65 (0.64-0.67)	0.80 (0.79-0.81)	0.73 (0.72-0.74)^b^	0.89 (0.89-0.90)

a *ICD-10*: *International Statistical Classification of Diseases, Tenth Revision*.

b Highest score for each scenario.

Among base-sized models, NorDeClin-BERT-base-NorICD outperformed all other base models in every scenario, with precision scores of 0.65 (all codes multilabel), 0.80 (top-5), 0.71 (top 80% most-used codes multilabel), and 0.89 (top 80% most-used codes top-5). This performance closely approaches that of the large models, further reinforcing the strength of domain-specific pretraining even with smaller architectures.

### Recall

Table 5 reports the recall scores across all models and evaluation scenarios. NorDeClin-BERT-large-NorICD consistently achieved the highest recall across all 4 evaluation settings, with scores of 0.48 (all codes multilabel), 0.82 (all codes top-5), 0.54 (top 80% most-used codes multilabel), and 0.88 (top 80% most-used codes top-5). The largest improvement was observed in the multilabel settings, where it outperformed the general-domain NorBERT3-large-NorICD by 5% points in all codes (0.48 vs 0.43) and 4 points in the top 80% codes (0.54 vs 0.50), underscoring the advantage of domain-specific pretraining at scale.

**Table 5.** Comparison of the recall of different BERT (Bidirectional Encoder Representations from Transformers) models.

Model size and model name	All codes, 95% CI		Top 80% codes, 95% CI	
	Multilabel	Top-5	Multilabel	Top-5
Base				
NorDeClin-BERT-base-NorICD^a^	0.45 (0.44-0.46)	0.81 (0.80-0.81)	0.51 (0.50-0.52)	0.87 (0.86-0.88)
SweDeClin-BERT-SweICD	0.25 (0.24-0.26)	0.59 (0.58-0.60)	0.29 (0.28-0.30)	0.65 (0.63-0.66)
SweDeClin-BERT-NorICD	0.41 (0.40-0.42)	0.78 (0.77-0.79)	0.48 (0.47-0.49)	0.85 (0.84-0.86)
ScandiBERT-NorICD	0.40 (0.39-0.41)	0.78 (0.77-0.79)	0.48 (0.47-0.49)	0.85 (0.84-0.86)
NorBERT3-base-NorICD	0.44 (0.43-0.45)	0.80 (0.79-0.81)	0.51 (0.50-0.52)	0.86 (0.86-0.87)
Large				
NorDeClin-BERT-large-NorICD	0.48 (0.47-0.49)^b^	0.82 (0.82-0.83)^b^	0.54 (0.53-0.55)^b^	0.88 (0.88-0.89)^b^
NorBERT3-large-NorICD	0.43 (0.42-0.44)	0.81 (0.80-0.82)	0.50 (0.49-0.51)	0.88 (0.87-0.88)^b^

a *ICD-10*: *International Statistical Classification of Diseases, Tenth Revision*.

b Highest score for each scenario.

Among the base-sized models, NorDeClin-BERT-base-NorICD also performed strongly, achieving 0.45 recall in all codes multilabel, 0.81 in top-5, 0.51 in top 80% most-used codes multilabel, and 0.87 in top 80% most-used codes top-5. It outperformed all general-domain baselines (ScandiBERT and NorBERT3-base), as well as the domain-specific Swedish models (SweDeClin-BERT-SweICD and SweDeClin-BERT-NorICD). Its recall closely matched or exceeded that of the larger NorBERT3-large-NorICD model in 3 of the 4 settings, further supporting the impact of domain-specific pretraining for improving recall in clinical coding tasks.

### *F*_1_-Score

Table 6 summarizes the *F*_1_-scores for all models across the 4 evaluation scenarios. NorDeClin-BERT-large-NorICD achieved the highest *F*_1_-score in all cases, with 0.54 for all codes multilabel, 0.81 for all codes top-5, 0.60 for the top 80% most-used codes multilabel, and 0.89 for top 80% top-5, consistently outperforming the general-domain NorBERT3-large-NorICD (0.50, 0.79, 0.58, and 0.88, respectively). The largest *F*_1_-score margin between the large models was observed in the all codes multilabel setting (0.54 vs 0.50), highlighting the impact of domain adaptation on balancing precision and recall in complex coding tasks.

**Table 6.** Comparison of *F*_1_-score of different BERT (Bidirectional Encoder Representations from Transformers) models.

Model size and model name	All codes, 95% CI		Top 80% codes, 95% CI	
	Multilabel	Top-5	Multilabel	Top-5
Base				
NorDeClin-BERT-base-NorICD^a^	0.52 (0.51-0.53)	0.79 (0.79-0.80)	0.58 (0.57-0.59)	0.88 (0.87-0.88)
SweDeClin-BERT-SweICD	0.27 (0.26-0.27)	0.55 (0.54-0.56)	0.31 (0.30-0.32)	0.63 (0.62-0.64)
SweClin-BERT-NorICD	0.46 (0.45-0.47)	0.76 (0.75-0.77)	0.54 (0.53-0.55)	0.86 (0.85-0.87)
ScandiBERT-NorICD	0.45 (0.44-0.46)	0.76 (0.75-0.77)	0.54 (0.53-0.55)	0.86 (0.85-0.87)
NorBERT3-base-NorICD	0.50 (0.49-0.51)	0.78 (0.78-0.79)	0.57 (0.56-0.58)	0.87 (0.86-0.88)
Large				
NorDeClin-BERT-large-NorICD	0.54 (0.53-0.55)^b^	0.81 (0.80-0.82)^b^	0.60 (0.60-0.61)^b^	0.89 (0.89-0.90)^b^
NorBERT3-large-NorICD	0.50 (0.49-0.51)	0.79 (0.78-0.80)	0.58 (0.56-0.59)	0.88 (0.87-0.89)

a *ICD-10*: *International Statistical Classification of Diseases, Tenth Revision*.

b Highest score for each scenario.

Among base-sized models, NorDeClin-BERT-base-NorICD also demonstrated strong performance with *F*_1_-scores of 0.52, 0.79, 0.58, and 0.88, respectively. It outperformed all other base models across every scenario and matched or exceeded the performance of the larger NorBERT3-large-NorICD in all 4 settings, further validating the strength of clinical domain pretraining even in smaller architectures.

### Interpretability

**Figure 5.**
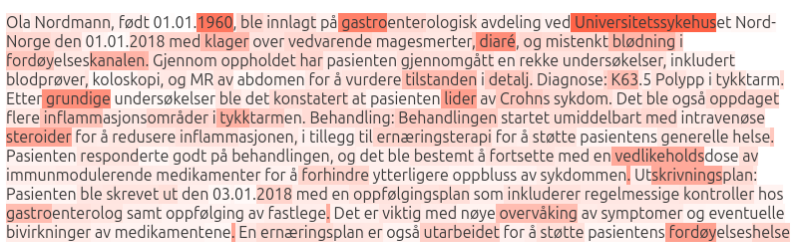
Attention distribution of NorDeClin-BERT-base-NorICD on a synthetic clinical text. A translation of the text is provided in Multimedia Appendix 1. BERT: Bidirectional Encoder Representations from Transformers; *ICD-10*: *International Statistical Classification of Diseases, Tenth Revision*.

Figure 5 illustrates the attention distribution of NorDeClin-BERT-base-NorICD in a synthetic clinical text. The attention appears to be distributed relatively uniformly throughout the clinical description, suggesting that the model focuses on a comprehensive contextual understanding of the text to make predictions. Key medical terms like diaré (diarrhea), blødning (bleeding), Crohns sykdom (Crohn disease), and inflammatorisk tarmsykdom (inflammatory bowel disease) receive high attention, indicating their importance in the model’s decision-making process. The model’s interpretability is based on its attention to clinical descriptions and terminology. This approach provides valuable insights into how the model processes natural language to arrive at its predictions. It is particularly useful in understanding how the model infers *ICD* codes from medical text alone, mimicking the process a human expert might follow when assigning codes based on clinical narratives.

**Figure 6.**
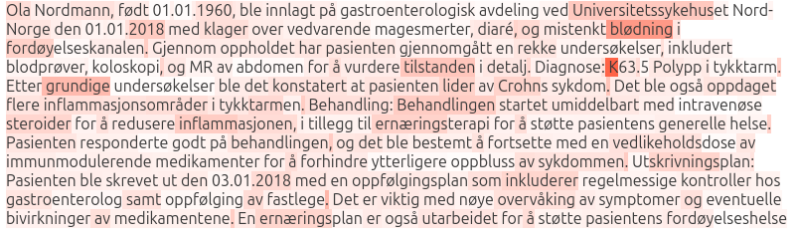
Attention distribution of NorDeClin-BERT-large-NorICD on the same synthetic clinical text. A translation of the text is provided in Multimedia Appendix 1. BERT: Bidirectional Encoder Representations from Transformers; *ICD-10*: *International Statistical Classification of Diseases, Tenth Revision*.

Figure 6 shows the attention distribution of NorDeClin-BERT-large-NorICD applied to the same synthetic clinical text. Like the base model, it assigns high attention weights to terms such as blødning, Crohns sykdom, and inflammatorisk tarmsykdom. However, the large model displays a slightly more focused and confident pattern, with attention concentrated more tightly around diagnostically relevant phrases. This may reflect the benefit of both greater model capacity and pretraining on the full corpus, resulting in more targeted representation learning.

Comparing the 2 models, both demonstrate strong interpretability by attending to clinically meaningful concepts. NorDeClin-BERT-large-NorICD appears to apply attention more selectively, in line with its superior classification performance. These visualizations support the idea that domain-specific pretraining not only improves predictive performance but also enhances transparency and trust in real-world clinical applications.

The distribution of attention across the text, focusing on key medical terms, suggests that the models have developed a nuanced understanding of clinical language. This method of interpretation allows us to understand which parts of the clinical narrative the models consider most relevant for predicting *ICD* codes. This ability to extract relevant information from various parts of the text indicates a robust and generalizable approach to *ICD* code prediction. It showcases the models’ capacity to process and understand clinical narratives in a way that aligns with human expert reasoning.

This interpretability analysis highlights the NorDeClin-BERT models’ potential to assist health care professionals and improve their trust by providing insight into the reasoning behind the predicted *ICD* codes. The models’ attention to a broad range of clinical terms and contexts suggests their potential adaptability to various types of medical narrative, which is crucial for real-world applications in diverse health care settings.

## Discussion

### Principal Findings

The development and evaluation of 2 variants of NorDeClin-BERT for *ICD-10* code classification tasks have yielded insightful results, highlighting their capabilities and potential applications in Norwegian health care settings. Both NorDeClin-BERT-base-NorICD and NorDeClin-BERT-large-NorICD have emerged as frontrunners, demonstrating higher accuracy, precision, recall, and *F*_1_-scores across both all codes and the top 80% most-used codes. These findings underscore the robustness and efficiency of the models in handling diverse and prevalent code classifications.

The good performance of the NorDeClin-BERT models, especially in the context of the top 80% most-used codes, suggests that these models have effectively captured the underlying patterns and nuances of the most frequent classifications in Norwegian clinical texts. This capability is critical in practical applications where prioritizing common codes can substantially enhance operational efficiency and accuracy. At the same time, the models, particularly NorDeClin-BERT-large, showed notable improvements in multilabel classification across both full and frequent-code scenarios, outperforming all baseline models in recall and *F*_1_-score. Furthermore, the high precision of the NorDeClin-BERT models indicates their utility in scenarios where the cost of false positives is high, making them an ideal choice for critical applications in medical coding and documentation.

An important aspect of our study is the comparison of models with different sizes and architectures. NorDeClin-BERT was developed in both base and large configurations, with the base model built on the BERT-base architecture (≈110 million parameters) and the large model using a BERT-large architecture (≈340 million parameters). NorDeClin-BERT-base consistently outperformed or matched the performance of other models, including the larger general-domain NorBERT3-large model. This finding is particularly noteworthy, as it challenges the common assumption that larger models invariably lead to better performance. The success of NorDeClin-BERT-base suggests that, for specialized tasks such as *ICD-10* coding in Norwegian clinical texts, a well-tuned base-size model can be highly effective and potentially more efficient in terms of computational resources and inference time.

The comparable or better performance of NorDeClin-BERT-base to larger models such as NorBERT3-large also highlights the importance of domain-specific pretraining and fine-tuning. It appears that the targeted approach of training on Norwegian clinical texts has allowed even the smaller NorDeClin-BERT variant to develop a more nuanced understanding of medical terminology and context, compensating for its reduced size. This observation has significant implications for model development in specialized domains, suggesting that carefully curated training data and domain-specific adaptation can be as important as, if not more important than, raw model size.

Furthermore, the efficiency of a smaller model like NorDeClin-BERT-base has practical advantages in clinical settings. It can be more easily deployed in environments with limited computational resources, potentially allowing for faster inference times and lower hardware requirements. This could facilitate broader adoption across various health care institutions, including those with constrained IT infrastructures.

The findings of this study have broader implications for the implementation of machine learning in Norwegian clinical settings. The NorDeClin-BERT models can substantially reduce the workload of health care professionals by automating routine coding tasks, allowing them to focus more on patient care and less on administrative duties. In addition, the enhanced accuracy and precision of these models can contribute to better patient outcomes by ensuring more accurate reporting and documentation, which, in turn, can lead to more targeted and effective patient care plans in Norwegian hospitals.

The attention-based interpretability analysis provides valuable insight into NorDeClin-BERT models’ decision-making process, which could enhance trust and adoption among health care professionals. The models’ ability to focus on relevant clinical terms when *ICD* codes are not present demonstrates their potential to generalize well to various clinical narratives.

Our study not only demonstrates the effectiveness of the NorDeClin-BERT models in *ICD-10* coding tasks but also provides valuable insights into the trade-offs between model size, domain-specific training, and performance in specialized NLP tasks. These findings could guide future research and development in clinical NLP, potentially leading to more efficient and effective AI solutions in health care.

### Broader Implications of *ICD-10* Coding Performance

While this study focuses on *ICD-10* coding for clinical documentation, structured coding also plays a crucial role in several other domains, including billing, epidemiological research, clinical registries, and decision support systems. In billing and insurance claims, accurate *ICD-10* coding ensures proper reimbursement and minimizes administrative errors. In epidemiological studies, these codes are essential for monitoring disease prevalence and public health trends, where high recall is particularly important to ensure comprehensive case identification and minimize underreporting. Similarly, clinical registries rely on structured diagnostic coding to maintain high-quality datasets, where both precision and recall influence the completeness and reliability of registry-based research. Additionally, in clinical decision support systems, *ICD-10* codes are often used to trigger alerts, inform risk assessments, or guide treatment recommendations, where high precision is crucial to avoid false-positive alerts that could contribute to alert fatigue and unnecessary interventions. While our study does not directly evaluate these applications, our findings suggest that models like NorDeClin-BERT-base-NorICD and NorDeClin-BERT-large-NorICD have the potential to improve coding accuracy in such contexts, thereby enhancing the quality of structured health data across multiple domains. Future research could explore domain-specific adaptations to optimize NLP-driven *ICD-10* coding for these different use cases.

### Limitations and Future Directions

While the results of this study are promising, several limitations must be acknowledged. First, the performance of the NorDeClin-BERT models might vary with different datasets or coding systems not covered in this study, particularly those outside the gastroenterology domain. This suggests the need for wider validation across various medical specialties and health care institutions in Norway to fully understand the generalizability of the findings.

Future research should aim to address these limitations by expanding the scope of the datasets and coding systems, potentially including other medical specialties and health care institutions across Norway. Exploring the integration of the NorDeClin-BERT models into real-world clinical workflows in Norwegian hospitals and assessing their impact on efficiency and patient care outcomes would provide valuable insights into their practical utility.

Furthermore, investigating the interpretability of the NorDeClin-BERT models and user trust in automated coding systems represents a crucial research area, as these factors greatly influence the adoption of AI technologies in health care. Developing explainable AI techniques tailored to the Norwegian clinical context could further improve the transparency and trustworthiness of these models, potentially accelerating their integration into Norwegian health care systems.

### Conclusions

This study introduced 2 versions of NorDeClin-BERT, domain-specific BERT models specifically developed for automating *ICD-10* code assignments from clinical notes within the Norwegian gastroenterological domain. By benchmarking these models against both general-domain and cross-lingual BERT baselines, we addressed 3 core RQs. First (RQ1), we found that domain-specific pretraining on Norwegian clinical text consistently improved *ICD-10* classification performance across all evaluation metrics, compared with general-domain Norwegian models and Swedish clinical models. Second (RQ2), we showed that scaling the model size from base to large further enhanced performance, most notably in multilabel scenarios, demonstrating that model capacity can amplify the benefits of domain adaptation. Third (RQ3), NorDeClin-BERT-base matched or outperformed NorBERT3-large in multiple scenarios, highlighting the value of targeted pretraining even with smaller architectures.

Compared with previous work on Swedish *ICD-10* classification using SweDeClin-BERT [15], our models achieved competitive or superior performance, especially under strict multilabel evaluation, despite differences in language and dataset structure. To our knowledge, this is the first study to develop and evaluate BERT-based models for *ICD-10* coding in the Norwegian language, setting a new benchmark for future clinical NLP research in this area.

Through detailed analysis of accuracy, precision, recall, and *F*_1_-score, our findings demonstrate the potential of domain-specific language models to support structured clinical documentation, reduce administrative burden, and enable more accurate downstream analytics in Norwegian health care. The results highlight the NorDeClin-BERT models as superior in terms of accuracy, precision, recall, and *F*_1_-score for both all codes and the top 80% most-used codes, consistently outperforming other BERT variants, including ScandiBERT, NorBERT3-base, NorBERT3-large, and SweDeClin-BERT. NorDeClin-BERT-large-NorICD demonstrated the highest overall performance, while NorDeClin-BERT-base-NorICD matched or exceeded the performance of larger general-purpose models in multiple scenarios. Both models demonstrate an improved ability to capture the nuances of the Norwegian language and the complexity of medical coding. The study also underscores the relevance of language-specific and domain-specific models, as evidenced by NorDeClin-BERT’s improved performance compared with models pretrained on general Scandinavian languages.

The attention-based interpretability analysis provided valuable insight into the NorDeClin-BERT models’ decision-making processes, demonstrating their ability to focus on relevant clinical terms and adapt to the presence or absence of explicit *ICD* codes in the text. This feature enhances the models’ potential for generalization and practical application in diverse clinical settings across Norway.

## Supplementary material

10.2196/66153 Multimedia Appendix 1 English translation of the text presented in Figure 5.

## Abbreviations

AI: artificial intelligence
BERT: Bidirectional Encoder Representations from Transformers
CAC: computer-assisted coding
GPU: graphics processing unit
*ICD*: *International Classification of Diseases*
*ICD-10*: *International Statistical Classification of Diseases, Tenth Revision*
ICD-O-3: International Classification of Diseases for Oncology
LIME: Local Interpretable Model-agnostic Explanations
MLM: masked language modeling
NLP: natural language processing
RQ: research question
SHAP: Shapley additive explanations
