# Supplementary material for: Domain-Specific Pretraining of NorDeClin-Bidirectional Encoder Representations From Transformers for International Statistical Classification of Diseases, Tenth Revision, Code Prediction in Norwegian Clinical Texts: Model Development and Evaluation Study
Source: JMIR AI. 2025 Aug 25;4:e66153. doi: 10.2196/66153 (PMC12377785; doi:10.2196/66153)
Supplement: Multimedia Appendix 1 [file ai-v4-e66153-s001.docx]

## Multimedia Appendix 1

Below is the English translation of the text presented in Figure 5, which provides a clinical case description in Norwegian:

*Ola Nordmann, born on 01.01.1961, was admitted to the gastroenterology department at* ***University Hospital Nord-Norge*** *on 01.01.2018 with complaints of persistent abdominal pain,* ***diarrhea****, and suspected bleeding in the* ***digestive tract****. During the hospital stay, the patient underwent several examinations, including* ***induced blood sampling, colonoscopy, and MRI of the abdomen to assess the condition in detail****. Diagnosis:* ***K63.5 Polyp in the colon****. After thorough examinations, it was found that the patient suffers from* ***Crohn’s disease****. Further,* ***four inflammatory lesions*** *were discovered in the colon. Treatment was immediately initiated with* ***intravenous steroids*** *to reduce inflammation, along with a* ***nutritional plan*** *to support the patient's general health. The patient was also prescribed medication, and due to the chronic nature of the disease, a* ***maintenance regimen*** *of immunomodulatory medications was introduced to prevent further flare-ups.* ***Discharge plan:*** *The patient was discharged on* ***01.03.2018*** *with a follow-up plan that includes* ***regular check-ups with a gastroenterologist and follow-up by a general practitioner****. It is essential to monitor symptoms and any potential side effects of the medications. A* ***nutritional plan*** *has also been developed to support the patient’s* ***digestive health****.*
